# Insights into earthquake hazard map performance from shaking history simulations

**DOI:** 10.1038/s41598-018-20214-6

**Published:** 2018-01-30

**Authors:** Kris Vanneste, Seth Stein, Thierry Camelbeeck, Bart Vleminckx

**Affiliations:** 1 Royal Observatory of Belgium, B-1180 Brussels, Belgium; 2 Department of Earth and Planetary Sciences, Northwestern University, Evanston, IL 60208 USA

## Abstract

Why recent large earthquakes caused shaking stronger than shown on earthquake hazard maps for common return periods is under debate. Explanations include: (1) Current probabilistic seismic hazard analysis (PSHA) is deficient. (2) PSHA is fine but some map parameters are wrong. (3) Low-probability events consistent with a map sometimes occur. This issue has two parts. Verification involves how well maps implement PSHA (“have we built the map right?”). Validation asks how well maps forecast shaking (“have we built the right map?”). We explore how well a map can ideally perform by simulating an area’s shaking history and comparing “observed” shaking to that predicted by a map generated for the same parameters. The simulations yield shaking distributions whose mean is consistent with the map, but individual shaking histories show large scatter. Infrequent large earthquakes cause shaking much stronger than mapped, as observed. Hence, PSHA seems internally consistent and can be regarded as verified. Validation is harder because an earthquake history can yield shaking higher or lower than the hazard map without being inconsistent. As reality gives only one history, it is hard to assess whether misfit between a map and actual shaking reflects chance or a map biased by inappropriate parameters.

## Introduction

Few issues in seismology have greater impact for society, and have generated more heated debate, than the question of how well earthquake hazard maps used as inputs to codes for earthquake-resistant construction predict future shaking^1–5^. Probabilistic seismic hazard analysis (PSHA), which has been used worldwide for almost 50 years, uses estimates of the probability of future earthquakes and the resulting shaking to predict the shaking expected with a certain probability over a given time^6,7^. However, questions about the method have been raised repeatedly^8–11^. The 2011 Tohoku, 2010 Haiti, and 2008 Wenchuan (China) earthquakes catalyzed discussions among seismologists and earthquake engineers about the fact that large earthquakes often cause shaking much higher than shown on earthquake hazard maps, and thus extensive damage and many fatalities. This situation has led to the interpretation that “the seismic crystal ball is proving mostly cloudy around the world”^12^.

Three general and overlapping explanations have been offered for this situation. In the first, “the hazard map and the methods used to produce it are flawed and should be discarded”^3^. It has been argued that it would be more useful to use deterministic methods that seek to predict the maximum shaking, rather than that expected with a given probability^13,14^. In the second, the PSHA method is fine but maps are biased by incorrect parameters and/or assumptions used in making them. For example, the Tohoku earthquake was much bigger than expected because it occurred on a much longer fault than was considered in the input model^1^. The third invokes bad luck — because the maps are probabilistic forecasts, one should recognize that unlikely events sometimes occur^5^.

The debate is heated both because of the stakes — policy decisions involving billions of dollars and thousands of lives — and because little is known about how hazard maps actually perform. Because major earthquakes and the resulting strong shaking are rare events in any one area, seismologists have only recently begun to develop methods of assessing map performance^15,16^. These face the challenge that the available data since hazard mapping began span too short a time period. Hindcasting using historical shaking data spanning hundreds or thousands of years circumvents this difficulty. Although this shows interesting discrepancies between predicted and observed shaking, it uses data that were available when the map was made and may be prone to biases including limitations of the historical record^17^.

Here, we take an alternative approach, by simulating the shaking history of an area and comparing the simulated shaking at many sites over time to that predicted by a hazard map generated for the same parameters using current PSHA software. This approach is based on the Monte Carlo simulation method^18–20^, which has been proposed as an alternative approach to the classical calculation of the hazard integral^6^.

These simulations give insight into how well a hazard map should describe the actual shaking that will occur in the future, in the ideal case that the map’s assumptions about earthquake occurrence and ensuing ground motion are correct. In other words, what performance can we expect from a hazard map in the ideal case that we know where earthquakes will occur, how often they will occur, how large they will be, and what shaking will result? In reality, all these quantities have significant uncertainties^21^, so a real map’s performance is likely to be poorer.

This approach lets us consider two aspects of assessing the performance of systems that seek to forecast future events. Verification involves assessing whether the system — typically computer code — implements the underlying conceptual model correctly. In this case, do hazard maps implement the PSHA algorithm correctly (“have we built the map right”)? Validation asks how well the resulting forecast describes what actually occurs — how well does a map forecast actual shaking (“have we built the right map”)? It is worth noting that meteorologists use “verification” for what we term “validation”, following systems engineers and hydrologists^22,23^. This paper starts out on the verification issue, by examining how the algorithm and code should be expected to behave. The resulting insights have important implications when considering validation, namely to assess how well a PSHA map for an actual area describes the shaking that actually occurred, which depends on both the algorithm and the specific model assumptions and parameters. Validation studies involve real data for specific areas, which is beyond the scope of this study. However, our quasi-realistic simulations give insights into how PSHA could be expected to work in the real world. In addition, they point the way to more detailed simulations that could be conducted for specific areas.

## Results

### Map performance over time

**Figure 1:**
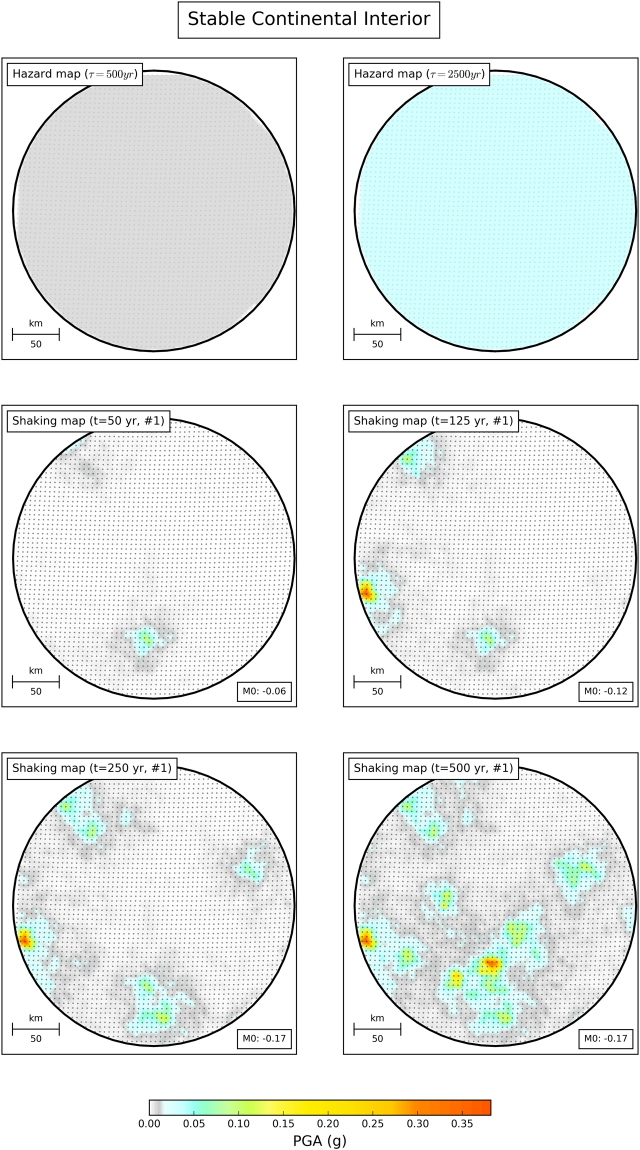
Comparison of hazard maps and shaking maps for model area with seismicity similar to a stable continental interior. Top row: Hazard maps for return periods of 500 and 2500 years. The maps are uniform across the area, because the expected level of shaking is the same. Middle and bottom rows: Maps of maximum shaking at each point after observation times of 50, 125, 250 and 500 years, for one simulation. M0 denotes misfit between the fraction of points where shaking exceeds that in the 500-yr hazard map, and the exceedance fraction expected for the corresponding observation time^30^.

**Figure 2:**
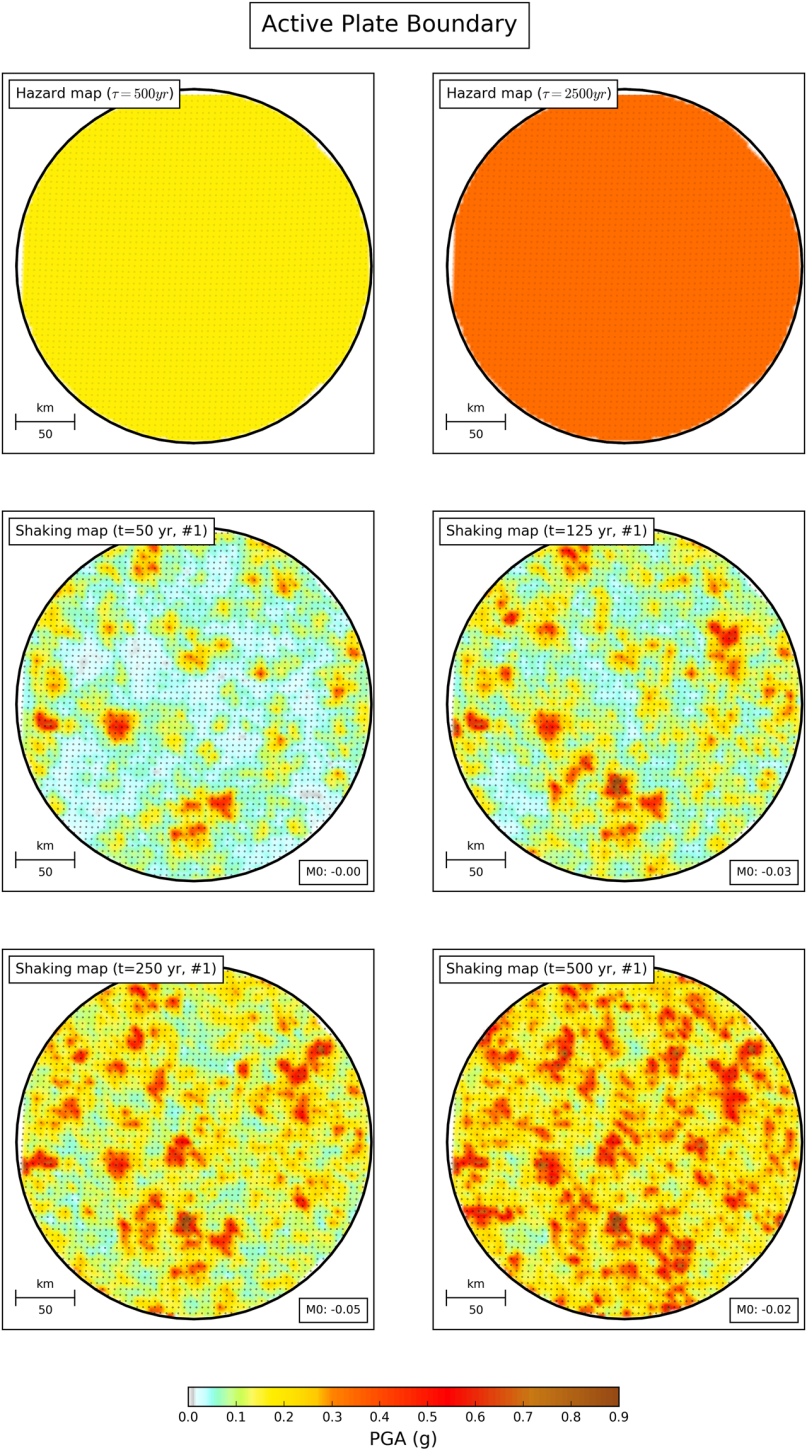
Comparison of hazard maps and shaking maps for model area with simulated seismicity similar to an active plate boundary. Panels are similar to Fig. 1.

We consider a hypothetical region (Figs 1 and 2) within which earthquakes occur randomly, with magnitudes given by a prescribed magnitude-frequency distribution (MFD). A ground motion prediction equation describes the resulting shaking in terms of a mean with specified uncertainty. We considered two activity rates: one corresponding to stable continental interiors like Northwest Europe and one 100 times greater, similar to a plate boundary like that in California. For each, we used OpenQuake open-source software^24^ to compute probabilistic seismic hazard maps with return periods of 500 and 2500 years. The resulting maps are confined to a smaller test area where hazard is uniform. The 2500-year map predicts higher hazard because larger earthquakes and higher shaking intensities are more likely to occur when longer time intervals are considered.

We then compute maps of simulated ground motion by generating 1000 random earthquake histories 2500 years long, assuming earthquakes occur uniformly on a grid of points 5 km apart, with magnitudes given by the MFD, at times obeying a Poisson distribution. For each synthetic earthquake, we simulate ten random ground-motion fields and archive the shaking at each grid point. This results in 10,000 ensembles of ground-motion fields (“shaking histories”), for each of which we produce maps of “observed” maximum shaking at each point after observation times of 50, 125, 250, 500, 750, 1000, 1500 and 2500 yr. The procedure is described in more detail in the Methods section.

As shown in Figs 1 and 2, some places experience shaking higher than on the hazard map, while others experience shaking lower than shown on the map. For example, after only 50 years some sites on the plate boundary experienced shaking stronger than shown on the 2500-year map. This matches experience that when large earthquakes happen, shaking is often much stronger than shown on hazard maps. For longer observation times, the number of such sites increases.

These exceedances do not necessarily invalidate the map, because PSHA maps predict the shaking that should be exceeded with a certain probability over a given time. At a point on the map, the probability *p* that during *t* years of observations shaking will exceed (at least once) a value expected once in a τ*-*year return period is assumed to be described by a Poisson distribution, *p* = *1* − exp(−*t*/τ)^7^. This probability is small for *t*/τ small and grows with observation time (Figs 1 and 2). Considering the ergodic assumption in PSHA^25^, the fraction of sites within a map at which observed shaking exceeds the mapped value should behave the same way^17,25–31^. Hence the shaking shown on a map with a τ-year return period should be exceeded at 10% of the sites in *t* = τ/10 years, 39% in *t* = τ/2 years, and 63% in *t* = τ years.

**Figure 3:**
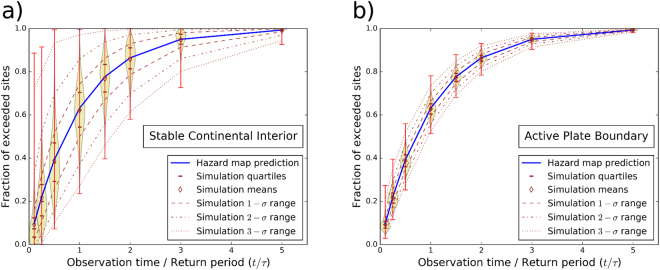
Comparison between expected and sampled fractions of sites where maximum shaking exceeds that in the 500-year hazard map as a function of observation time: (**a**) simulations for model area with seismicity comparable to stable continental interiors; (**b**) simulations for model area with seismicity comparable to active plate boundaries. The means and medians of the 10,000-simulation ensemble are consistent with those predicted, and the ensemble is concentrated about them with some scatter. The scatter decreases for longer simulations and is less for the more active plate boundary area.

Figure 3 shows the distribution of exceedance fractions for the 10,000 simulations compared to those predicted by the map with 500-year return period. The means and medians of this ensemble are consistent with those predicted. This is also consistent with earlier studies performing Monte Carlo simulations for a single site^18–20^, showing that the fraction of samples in the distribution of maximum shaking (in a particular timespan) that exceed a given hazard level converges to the Poisson probability if the number of samples is sufficiently large. This equivalence between the mean exceedance fraction over space and the limit of the exceedance fraction over a number of samples (~time) at one site is the expression of ergodicity^25^, which is due to the spatial uniformity of our model. More important for our purposes, however, is that the ensemble has considerable scatter about the mean. For example, after 50 years for the stable region (*t*/τ = 0.1) the mean is 0.1, as expected, but the fraction of exceeded sites varies from essentially 0 to 0.9. The scatter decreases for longer simulations (increasing t/τ), because as observation time increases, the largest earthquakes and resulting shaking are increasingly likely to have occurred. For the same reason, scatter is much less for the more active plate boundary, although it would be larger if we had extended the MFD to higher magnitudes.

It is important to note that the variability of simulated exceedance fractions we demonstrate for a region with many sites should not be confused with the variability in maximum shaking for an individual site, presented in previous simulations^19^. In terms of exceedance fraction, the latter represents only one value. Thus, in contrast to the mean value, the variability of spatial exceedance fractions represents another dimension of the same underlying uncertainty that is not ergodic. The shape and width of this distribution (and the way these change with activity rate or more correctly MFD a-value) cannot simply be inferred from the variability in maximum shaking at one site. We also find that the variability of exceedance fractions is quasi-independent of the GMPE uncertainty truncation level (including zero), which is not the case for the single-site distribution of maximum shaking.

### Exceedance as a function of magnitude

**Figure 4:**
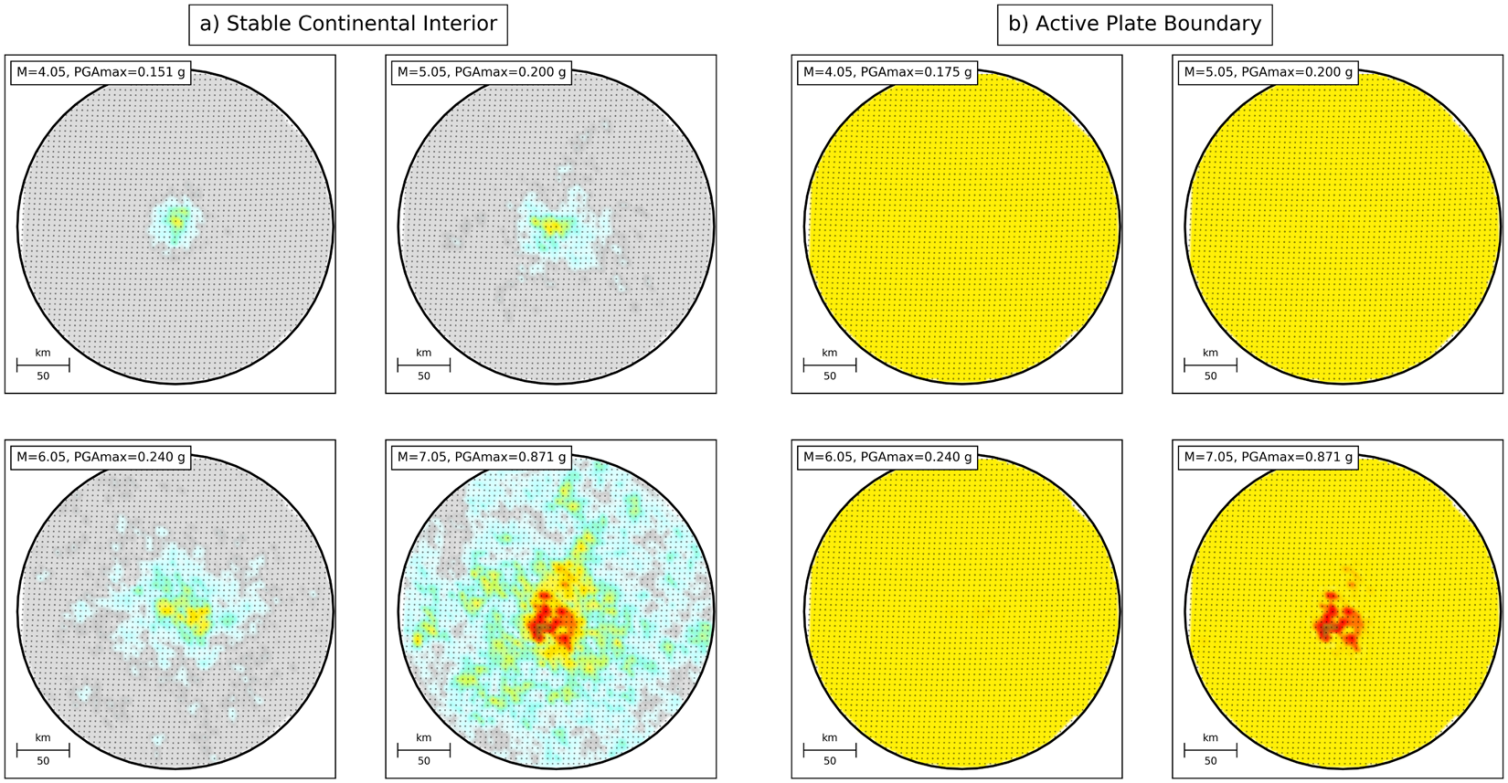
One random realization of ground-motion maps for earthquakes with magnitudes of 4.05, 5.05, 6.05 and 7.05 at the centre of the study area, superposed on the 500-yr hazard maps: (**a**) stable continental interior; (**b**) active plate boundary. Colour scale same as in Figs 1 and 2.

To assess the dependence of exceedance fractions on earthquake magnitude, we first compute ten random ground-motion maps for all possible earthquake locations and magnitudes in our model (~350,000 possible combinations, see Methods section). Figure 4 shows four of these maps, giving ground motion for earthquakes with different magnitudes at the same epicentral location. The shaking is superposed on the 500-year hazard maps for the stable continental interior (SCI) and active plate boundary (APB) cases, highlighting where exceedances occur. For the SCI, where the mapped hazard is low, even a magnitude 4.05 event produces exceedances at some sites. Larger earthquakes produce exceedances at more sites. However, for the APB, few or no exceedances are caused by magnitudes up to 6.05, while those produced by a magnitude 7.05 event cover a larger, but still relatively small, portion of the study area.

**Figure 5:**
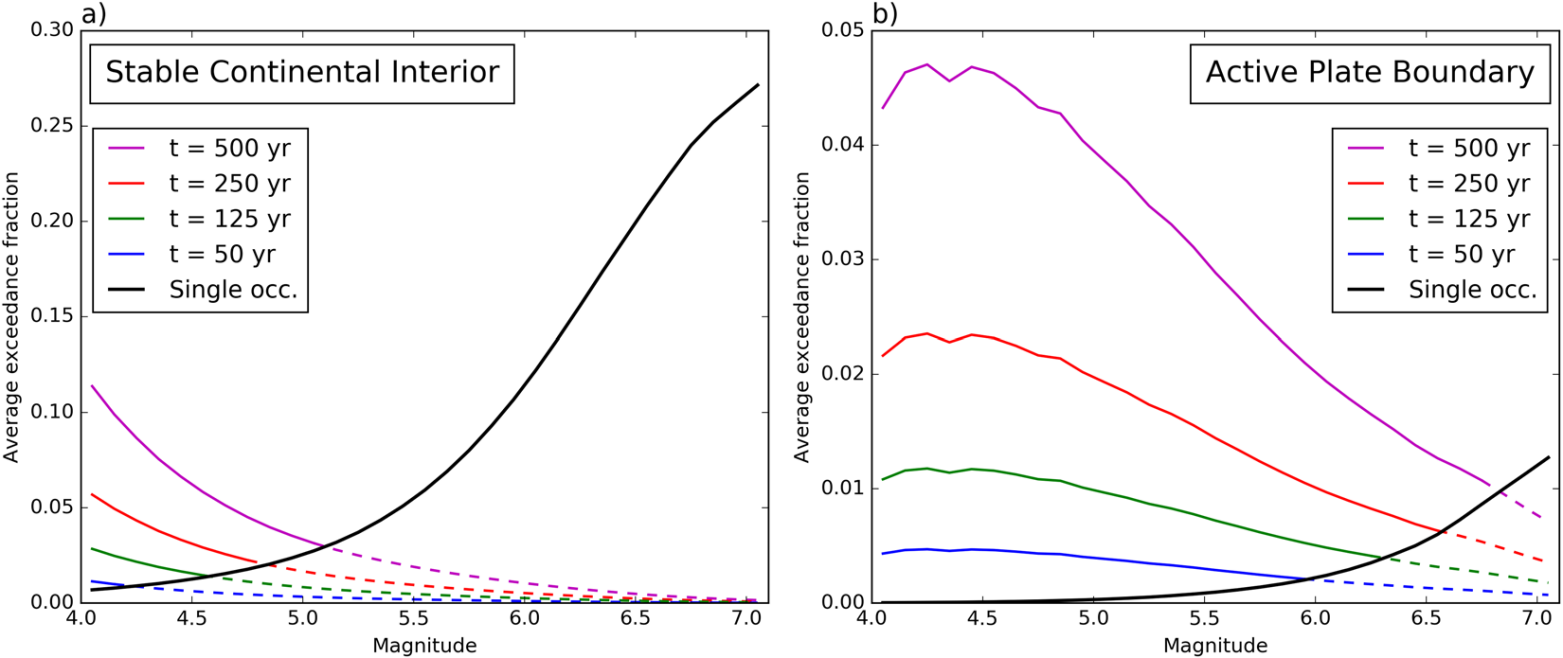
Exceedance fractions with respect to the 500-year hazard maps as a function of magnitude: (**a**) stable continental interior; (**b**) active plate boundary. Black lines (denoted as “Single occ.”) represent the average exceedance fraction produced by single occurrences of a given magnitude. Coloured lines correspond to cumulative exceedance fractions caused by all occurrences of a given magnitude over different time intervals (i.e., the black curve multiplied by the magnitude-frequency distribution and the observation time). Dashed parts of these curves correspond to magnitude interval where average number of occurrences in the corresponding observation time is below 1. In these intervals, average exceedance fractions for single occurrences (black curve) are higher than the cumulative exceedance fractions. The small notch in the cumulative curves for the APB case is likely due to insufficient random sampling. Note that the total exceedance fraction in the histograms is larger than the mean in Fig. 3, because multiple exceedances at the same site due to different events are counted only once in the latter.

Using such maps, we can compute the exceedance fraction with respect to the 500-year hazard maps as a function of magnitude for any epicentral location. Repeating this for all epicentral locations yields the average exceedance fraction due to a single occurrence of each considered magnitude, shown in Fig. 5 (black curves). These histograms show that the average exceedance fraction rises more strongly with magnitude for SCI compared to APB. An M = 6.05 earthquake on average causes exceedance in ~12% of the sites in the SCI case, but only in ~0.24% of the sites in the APB case. These differences simply reflect the fact that the hazard in SCI is lower compared to APB.

Finally, we assess the cumulative effect of all earthquakes of a particular magnitude over different observation times. We multiply the average exceedance fractions for a single occurrence by the number of occurrences in each magnitude bin, to obtain average cumulative magnitude – exceedance fraction histograms for different observation times, shown in different colours in Fig. 5. These histograms are equivalent to deaggregation by magnitude in PSHA, except that they apply to the whole map rather than just one site. The results vary as a function of magnitude differently between the SCI and APB cases. For the SCI case, the cumulative exceedance fractions decrease monotonically with increasing magnitude. This decrease indicates that the larger number of smaller earthquakes outweigh the higher exceedance fraction for larger magnitudes. Thus, the smallest magnitudes (M = 4.05) collectively cause the largest amount of exceedance, more than 10% for an observation time of 500 years (percentages mentioned hereafter are for the same observation time). For the APB case, cumulative exceedance fractions are still highest for low magnitudes, but the values (~4.5%) are lower compared to SCI, and they remain more or less at the same level up to M~5.0, beyond which they gradually drop to <1% for M = 7.05. Interestingly, the cumulative exceedance fractions caused by M = 6.05 earthquakes are not very different for the SCI (~1%) and APB (~2%) cases. However, for the SCI case, this is far below the exceedance fraction that would be caused on average by a single occurrence of such magnitude (black curve in Fig. 5). In other words, magnitudes that occur less than once on average in a given time interval (dashed interval in Fig. 5) cause unexpectedly large exceedances (i.e., larger than the exceedance probability of the hazard map) if they do occur. The magnitude above which the predicted number of occurrences drops below 1 is significantly lower in SCI compared to APB, and is lower for shorter observation times. In addition, average single-event exceedance fractions grow faster with increasing magnitude. Hence, in stable continental interiors many of the exceedances are caused by smaller-magnitude earthquakes, but infrequent large ones may cause very large exceedances. For active plate boundaries, the differences between different magnitudes are less pronounced, and individual occurrences of the highest magnitudes generally do not cause exceedance fractions far in excess of the cumulative ones. However, this would probably be different if we had assumed a higher maximum magnitude for this case. These different contributions as a function of magnitude demonstrate that the scatter of simulated exceedance fractions is primarily due to the rare occurrence of larger earthquakes, and explain why this scatter is larger for SCI compared to APB, and for short observation times compared to longer ones (Fig. 3).

### Implications for hazard-map validation

**Figure 6:**
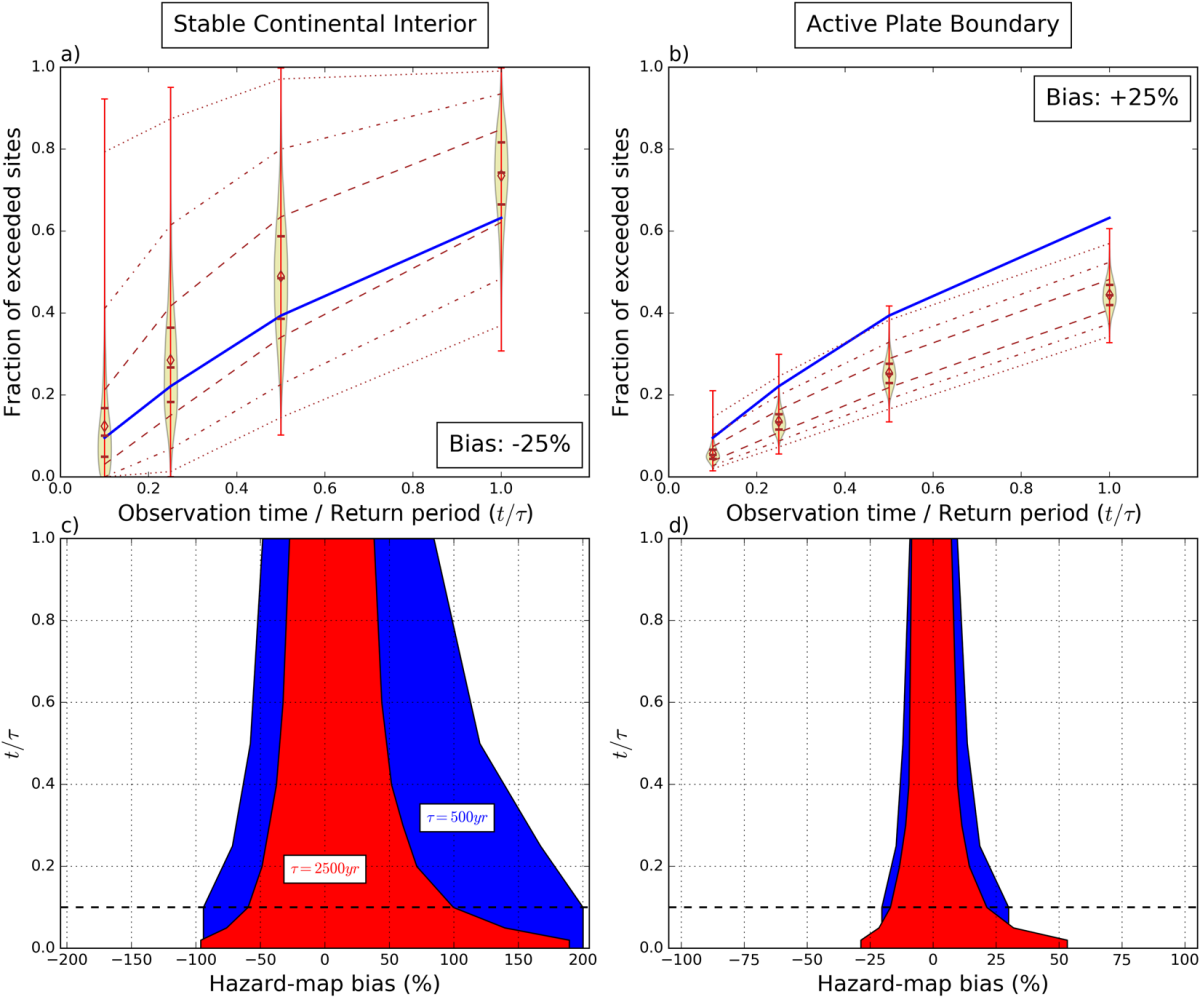
Limitations in detection of hazard-map bias based on the fractional exceedance metric. Top panels – Distribution of sampled vs. predicted exceedance fractions compared to a biased hazard map with 500-yr return period: (**a**) stable continental interior, bias = −25%; (**b**) active plate boundary, bias = +25%. Legend as in Fig. 3. Bottom panels – Range of hazard-map bias that cannot be detected at 2-sigma confidence level as a function of *t*/τ: (**c**) stable continental interior; (**d**) active plate boundary. Horizontal dashed line corresponds to *t*/τ = 0.1.

Our results have important consequences for assessing hazard-map performance based on misfits in fractional exceedance, and for exploring whether such misfit arises by chance or reflects a bias in the map. Given the large scatter of simulated exceedance fractions, we ask how much hazard-map bias could be detected for this ideal case at a given confidence level. To address this question, we first compute the distribution of exceedance fractions with respect to 500-yr hazard maps that are biased by adding or subtracting a fixed percentage. Figure 6 (top panels) shows an example for 25% bias. The resulting exceedance fractions are shifted upward and downward, respectively, relative to those predicted. However, due to the large scatter, a considerable period of observation is needed before the biased exceedance fractions become statistically different from that predicted. For the APB case, the Poisson curve intersects the two-sigma bound already near *t*/τ = 0.15, whereas for the SCI case, it is only near *t*/τ = 1 that the one-sigma bound is intersected.

In a second step, we repeat this procedure for a range of bias percentages, and determine the percentile of the predicted exceedance fraction for a particular time span in the sampled distribution. We then interpolate the bias corresponding to specific confidence intervals for different observation times. The results for 2-sigma confidence interval are shown in Fig. 6 (bottom panels). For the SCI case and an observation time of 50 years (dashed horizontal line in Fig. 6), the shortest time span tested and the one that is most appropriate for hazard-map validation, it is not possible to demonstrate at 2-sigma confidence level whether a hazard map with 500-yr return period underestimates or overestimates the actual hazard by 93% and >200%, respectively. The bias range that cannot be detected at 2-sigma confidence level becomes narrower with increasing activity rate. For the APB case, it is between approximately −20% and +30%. It should again be noted that the latter range is too optimistic, and would be wider if we had used a larger Mmax for the APB case.

It is also interesting to note that our finding that the scatter of exceedance fractions for a region with many sites decreases with increasing MFD a-value (Fig. 3) contrasts with simulation studies for a single site^32^, which demonstrated that the observation time window required to estimate with a given uncertainty the occurrence rate of ground motion having a particular return period is independent of the seismicity level. This indicates that our chances to validate hazard for a hazard map improve with increasing seismicity level (or area size), but not for a single site. Our results are for a test area of ~70,000 km², so increasing the size of area sources in the PSHA input model and/or of the region over which the hazard map is tested should improve our ability to validate hazard maps against observed shaking data based on the exceedance-fraction metric.

## Discussion

Our simulation results corroborate earlier conclusions that the probabilistic method should work as expected^33^, provided that all underlying assumptions about future earthquake occurrence and ensuing ground motion are correct. Hence, the PSHA algorithm as implemented in commonly used software appears to be internally consistent and can be regarded as verified. However, the large scatter revealed by our results implies that validation is more complicated because, even though many shaking histories are likely to be similar to a map’s prediction, some can yield exceedance fractions much higher or lower than predicted while being consistent with the model of seismicity underlying the hazard map. Moreover, a real map involves assumptions about more complicated source geometries and occurrence rates, which are unlikely to be exactly correct (or may even be inadequate) and thus will contribute additional scatter. Hence in the real world, with only a single earthquake shaking history for any area, it is hard to assess whether a misfit between actual shaking and a map — notably higher-than-mapped shaking — arises by chance or reflects biases in the map. This assessment is easier for more active (or larger) areas (Fig. 3). Analysis of the contribution of earthquakes with different magnitudes to exceedance of the hazard map shows that magnitudes that occur on average less than once in the life time targeted by a hazard map (i.e., the time span corresponding to its quoted exceedance probability) cause unexpectedly large exceedances if they do occur. This is particularly the case for less active areas, where the magnitude above which events occur less than once is significantly lower, and exceedance fractions produced by a single event grow faster with increasing magnitude.

This issue reflects a fundamental challenge for probabilistic forecasts. Forecasts of the probability of a range of values are increasingly used in applications including meteorology, finance, demography, and sports because they attempt to reflect the uncertainties in knowledge of the system. Because they allow low-probability extreme events, such events need not demonstrate weakness in the model. For example, when the spring of 2012 was the wettest on record in Britain despite being forecast as dry, the Meteorological Office admitted that its forecast was “not helpful” but likened it to the guide to a horse race — “any of the outcomes could occur, but some are more likely than others”^34^.

**Figure 7:**
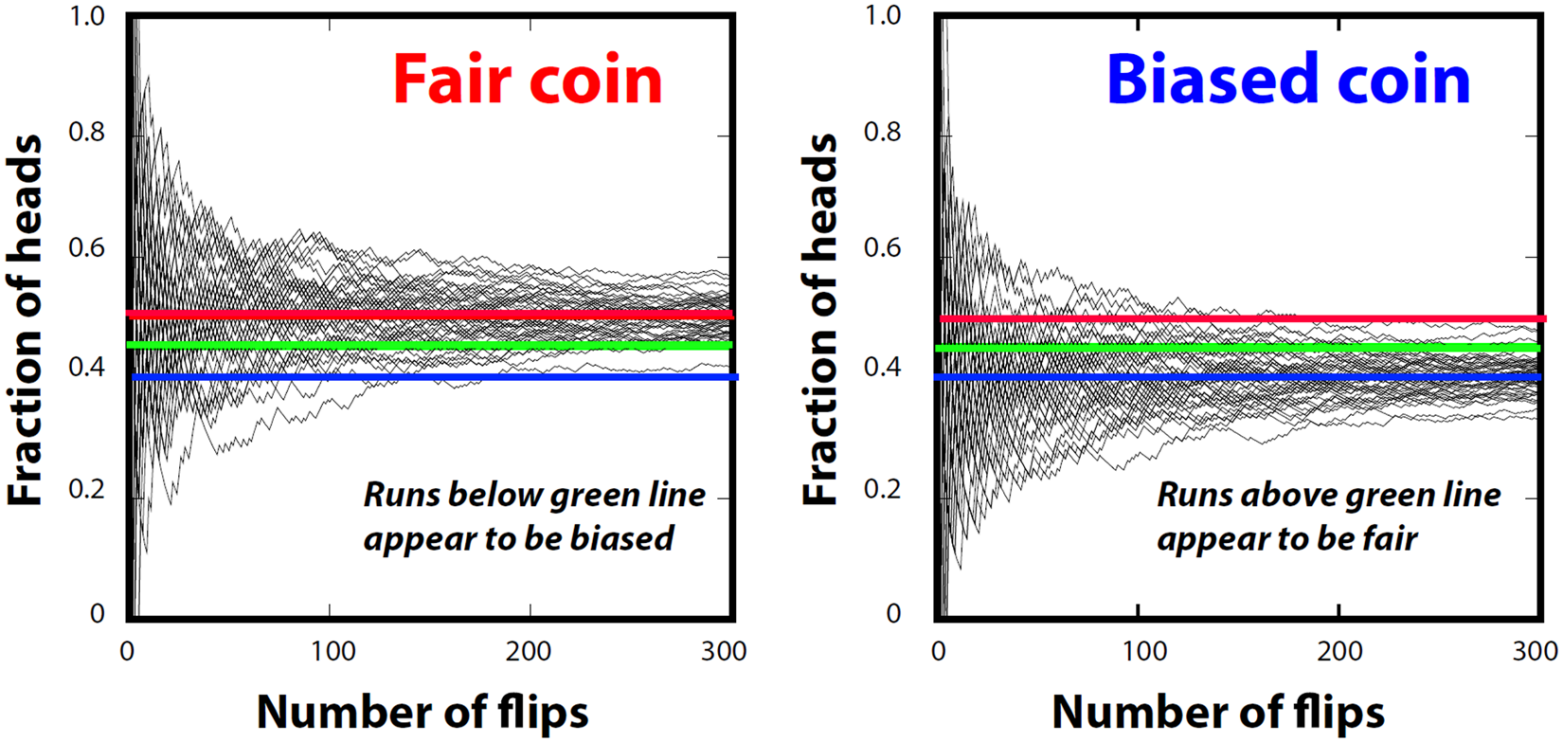
Demonstration of observation length needed to detect bias. Plots show fraction of heads resulting from a sequence of flips of a fair coin (left panel), which trends to 0.5 (red line), and a biased coin (right panel), which trends to 0.4 (blue line). Some runs look biased for a fair coin; some look fair for a biased coin. A single sequence has to be very long to convincingly distinguish fair from biased.

Assessing whether an earthquake hazard map performed poorly because of problems with the map or bad luck is analogous to trying to tell if a coin is fair — equally likely to come up heads or tails when flipped — or biased. Some sequences look biased for a fair coin; some look fair for a biased coin (Fig. 7). The mean of an ensemble of runs converges on the expected value with smaller standard deviation as the run gets longer. A single sequence has to be very long to convincingly distinguish fair from biased.

In summary, the fact that large earthquakes produce shaking much stronger than shown on hazard maps can be consistent with the predictions of probabilistic maps, and need not indicate that the maps are biased. In some cases, like the 2011 Tohoku earthquake^1^, the stronger-than-expected shaking reflects a poor choice of hazard map parameters. In other cases, there is no way to tell with the short periods of observations typically available whether they result from a bad map or bad luck. Due to this problem — which affects probabilistic forecasts in many applications — there are limits to how well we can expect hazard maps to predict future shaking, as well as to our ability to validate a given hazard map based on available observations, particularly when small and/or low-activity regions are concerned. Moreover, at this time, we do not know enough about map performance in real cases to have agreement about how good (or bad) an agreement between the predicted and actual performance of hazard maps should be required for them to be considered “valid” (or “invalid”). Hence, debate about the validity of model assumptions in actual hazard maps, as well as the utility of PSHA in general — which involves scientific, economic, and policy issues^35–37^ — is not easily resolved and will likely continue. Considering the large uncertainties, it would also be useful to carefully consider what level of detail is appropriate in our input models, as well as in the resulting hazard maps.

## Methods

**Figure 8:**
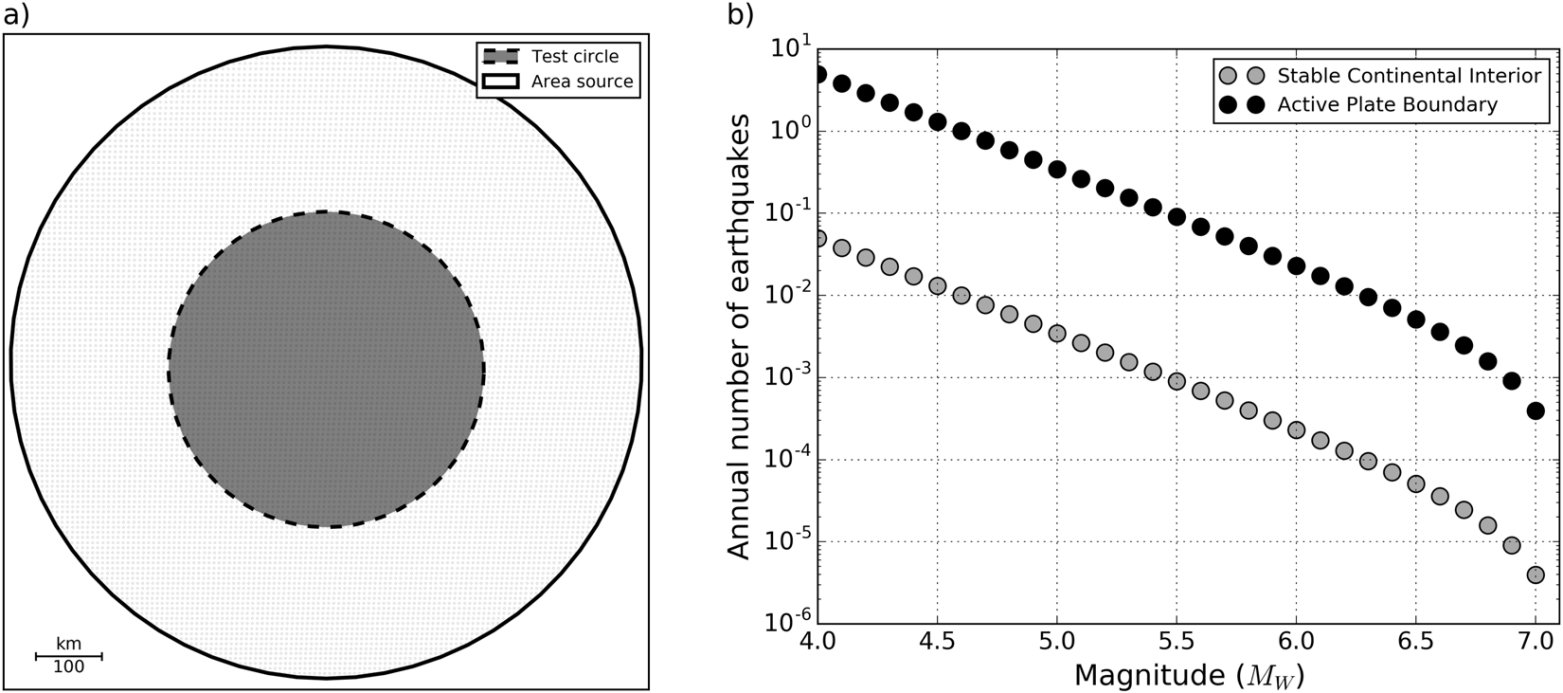
Model setup: (**a**) Geometry of the simulation region. Earthquakes occur within the larger circle and fractional exceedance is calculated at sites in the inner circle, to avoid edge effects; (**b**) Truncated Gutenberg-Richter magnitude-frequency distribution (MFD) between minimum and maximum magnitudes of 4.0 and 7.1, with a b-value of 0.975 and magnitude bin width of 0.1. We considered two very different activity rates, one corresponding to the average activity in stable continental regions and one for a plate boundary region, 100 times higher. Activity rates shown are normalized to 100,000 km² area.

We use a simplified area-source model (Fig. 8a) to compute hazard maps and simulated shaking maps. The model consists of a single circular area source with 300 km radius characterized by uniform seismicity following a truncated Gutenberg-Richter magnitude-frequency distribution (MFD) between minimum and maximum magnitudes of 4.0 and 7.1, with a b-value of 1.156 and magnitude bin width of 0.1. We considered two very different activity rates (Fig. 8b), corresponding to the average activity in stable continental Europe and a 100 times more active plate boundary area. The respective a-values (normalized to 100,000 km² surface area) are 2.32^38^ and 4.32. The latter is comparable to a-values reported for the larger California region^39^. The area source is discretized into a regular grid of points with 5 km spacing. These points correspond to the midpoints of finite ruptures modelled in the hazard computation and the sites where hazard and ground motion are computed. The integration distance, i.e. the maximum source-to-site distance used in the computations, was set to 150 km, i.e. half the radius of the area source.

Identical rupture and ground-motion parameters are used to calculate the hazard and ground-motion maps. All ruptures are modelled with strike, dip and rake of 0°, 45° and −90°, centred at a depth of 10 km, confined between upper and lower seismogenic depths of 0 and 20 km, with area scaled to magnitude following an empirical relation^40^ and an aspect ratio of 1. We selected a ground-motion prediction equation (GMPE)^41^ and a single ground-motion intensity measure, peak ground acceleration (PGA). We applied a GMPE uncertainty truncation level (expressed as number of standard deviations) of 3. The lowest PGA value resolved by the hazard map is 0.001 g.

To generate synthetic shaking maps, we first draw 1000 random earthquake histories over a time interval of 2500 yr with inter-event times following an exponential distribution with mean recurrence time and magnitude given by the different bins of the area-source MFD. Epicentral locations are randomly sampled from the same grid of points used to discretize the area source. For each synthetic earthquake, we simulate 10 random ground-motion fields with uncertainty range corresponding to the selected GMPE truncation level. For each activity rate, we thus obtain 10,000 ensembles of ground-motion fields, for each of which we construct maximum shaking maps after observation times of t = 50, 125, 250, 500, 750, 1000, 1500 and 2500 years (each interval including that preceding it in the list).

We compare the simulated maximum shaking maps and hazard maps by counting the number of sites where ground motion predicted by the hazard map is exceeded. To avoid edge effects, this fractional-exceedance comparison is restricted to a smaller concentric circle with radius corresponding to the integration distance (Fig. 8a). The total number of ground-motion sites is 11289, 2813 of which are inside the inner circle. All computations were performed using Python code we developed based on the oq-hazardlib library of OpenQuake^24^.

### Data Availability

The simulated data generated during this study are available from the corresponding author on request.
